# Relationship between the Presence of Human Papillomavirus in the Female Urethra and Recurrent Urinary Tract Infections

**DOI:** 10.3390/jcm13175329

**Published:** 2024-09-09

**Authors:** Cristina Mena-Ruiz, Julius Jan Szczesnieski, Magaly Márquez-Sánchez, Bárbara-Yolanda Padilla-Fernández, Javier Flores-Fraile, María-Fernanda Lorenzo-Gómez

**Affiliations:** 1 Department of Urology, University Hospital of Araba, 01009 Álava, Spain; 2 Department of Urology, University Hospital of Getafe, 28905 Getafe, Spain; 3 Renal Urological Multidisciplinary Research Group (GRUMUR), Institute of Biomedical Research of Salamanca (IBSAL), 37007 Salamanca, Spain; magalymarquez77@gmail.com (M.M.-S.);; 4 Department of Urology, Surgery Department, University of La Laguna, 38200 Santa Cruz de Tenerife, Spain; padillaf83@hotmail.com; 5 Surgery Department, University of Salamanca, 37007 Salamanca, Spain; 6 Department of Urology, University Hospital of Salamanca, 37007 Salamanca, Spain

**Keywords:** recurrent urinary tract infections, rUTIs, urethral pain, human papillomavirus, HPV

## Abstract

**Introduction:** Recurrent urinary tract infections (rUTIs) are highly prevalent health issues among women, significantly impacting their quality of life. Urethral pain or urethritis can arise from infectious or non-infectious origins. The presence of Human Papillomavirus (HPV) in the urogenital tract has been associated with high-risk sexual behaviors, but its presence in the female urethra without such behaviors has not been thoroughly investigated. **Objectives:** The study aims to determine the presence of HPV in the urethra of women with recurrent urinary tract infections (rUTIs) and concomitant urethral syndrome and to compare the clinical and microbiological characteristics of women with and without urethral HPV, specifically focusing on those without high-risk sexual behaviors. **Methods:** This prospective multicenter study included 138 women over 18 years old with rUTIs and concomitant urethral pain syndrome. High-risk sexual behaviors, sexually transmitted infections, and vaginitis were set as exclusion criteria. Participants were divided into two groups: NHPV (n = 72) with no urethral HPV and HPV (n = 66) with urethral HPV presence. Variables analyzed included age, body mass index (BMI), follow-up duration, comorbidities, treatments, toxic habits, surgical history, main symptoms, urine sediment findings, and cultures from urine and vaginal exudate. HPV genotyping was also performed. Descriptive statistics were used, along with Student’s *t*-test, Chi-square, Fisher’s exact test, ANOVA, and multivariate cluster analysis. **Results:** The NHPV group was older on average (48.75 years) compared to the HPV group (39.09 years). The HPV group had a longer follow-up period (2634 days vs. 1975 days in NHPV). Urinary incontinence was significantly more common in NHPV (63.89%) compared to HPV (18.18%) (*p* = 0.001). HPV-positive women had a higher prevalence of verrucous lesions in the vaginal introitus (64% vs. 8% in NHPV). Additionally, the HPV group showed higher rates of pyuria (27.27%), vaginal Candida albicans (36.26%), and positive urine cultures for *Escherichia coli* (47.83%), *Enterococcus faecalis* (36.36%), and *Klebsiella pneumoniae* (21.74%). No significant differences were observed between the groups concerning BMI, smoking habits, diabetes, or the main symptom at consultation. The most common HPV genotypes identified were G35, G42, and G66 (each 27%). Multivariate analysis revealed that sensitivity to nitrofurantoin was the most significant variable in the HPV group (importance of 0.96), followed by fosfomycin (0.79), trimethoprim (0.79), and amoxicillin (0.71). **Conclusions:** HPV was present in the urethra of 47.8% of women with rUTIs and concomitant urethral syndrome who did not exhibit high-risk sexual behaviors. These women were younger and had a longer duration of symptoms compared to those without urethral HPV. The identification of Enterococcus faecalis, Escherichia coli, and Klebsiella pneumoniae was more common in the HPV group. The sensitivity of bacteria to nitrofurantoin and fosfomycin is crucial for the clinical management of these patients. The presence of urethral HPV should be considered in the evaluation and treatment of women with rUTIs and urethral syndrome.

## 1. Introduction

Human Papillomavirus (HPV) is one of the most common sexually transmitted viruses, encompassing both oncogenic (high- and low-risk) and non-oncogenic variants [1,2]. HPV 16 is the most common oncogenic variant, detected in 20% of all HPV cases. A recent meta-analysis revealed a prevalence of 49% for any type of HPV and 35% for high-risk HPV in men [2,3].

HPV has been extensively studied in the female gynecological field, and its relationship with cervical cancer development is clear, particularly with oncogenic variants like HPV 16 and HPV 18 [2]. Other related cancers include laryngeal carcinoma, penile carcinoma, and anal carcinoma [4]. However, the etiological role of HPV infection in the pathogenesis of the urinary tract has not been clarified. HPV infections are known to occur frequently in external genitalia through sexual contact but can also occur in the urinary tract, including the urethra and bladder [4].

Since 1994, efforts have been made to establish a relationship between HPV and urethral cancer, with HPV detected in up to 59% of invasive and in situ carcinoma cases. The most frequent type was HPV 16 in 47% of invasive carcinoma cases [5]. Analysis of urinary tract biopsies in women with recurrent urethritis and cystitis showed an association with HPV 16 and the presence of urothelial metaplasia using polymerase chain reaction (PCR) [6].

Recurrent urinary tract infections (rUTIs) are a significant health problem, negatively affecting patients’ quality of life, social and sexual relationships, self-esteem, and work capacity [2]. Treatment sometimes involves antibiotics, potentially leading to resistance. Other accepted treatments include autovaccines [7]. However, the role of HPV in chronic inflammation and urinary symptoms in these patients is unknown [6].

Urethral pain or urethritis can have infectious or non-infectious origins, presenting with urinary tract symptoms, and must be distinguished from UTIs. They have been associated with high-risk sexual behaviors, similar to HPV [2], but have not been investigated in women without high-risk sexual behaviors.

The aims of this study are to establish the presence of Human Papillomavirus (HPV) in the urethra of women with recurrent urinary tract infections (rUTIs) and concomitant urethral syndrome and to describe the characteristics of the patients and bacteria, comparing women with urethral HPV to those without urethral HPV.

## 2. Methods

We conducted a prospective multicenter study of 138 women. Inclusion criteria: women over 18 years old with recurrent urinary tract infections (rUTIs) and concomitant urethral pain syndrome. Exclusion criteria: minors without informed consent, high-risk sexual behaviors, sexually transmitted diseases, vaginitis. The study was conducted from 1 January 2008 to 1 January 2023, with patients recruited from routine clinical practice.

rUTI was defined as the recurrence of complicated or uncomplicated urinary tract infections with a frequency of more than 3 episodes/year or 2 in the last six months with positive culture when symptomatic [2]. Urethral pain syndrome was defined as a condition characterized by pain referred to the urethra, reported by patients during anamnesis, physical examination, presence of purulent discharge from the meatus, more than 10 leukocytes per field in the absence of UTI, or gram stain with >5 polymorphonuclear cells/field in direct examination of urethral discharge [2].

Once included in the study, patients underwent HPV determination by urethral exudate (special cervical brush in urethra exudate-collection medium) in University Hospital of Salamanca. The sample was collected during consultation after diagnosing urethral pain along with rUTI presence. Two groups were defined based on the results: NHPV group (no HPV) and HPV group (presence of HPV). Epidemiological and clinical data were collected: age, body mass index (BMI), comorbidities, surgical history, main symptom, urine sediment findings, urine culture results causing UTIs with corresponding antibiograms, vaginal exudate results, and HPV genotypes in both exudates in the HPV group.

All procedures were conducted in accordance with the Declaration of Helsinki and Spanish legislation. The study protocol was reviewed and approved by the Research Ethics Committee with Medicines of Salamanca (approval code 202202932). Informed consent was obtained from each participant.

Results were analyzed using descriptive statistics, Student’s *t*-test, Chi-square, Fisher’s exact test, ANOVA (with Scheffe’s test for normal samples and Kruskal–Wallis for other distributions), Pearson’s and Spearman’s correlation studies, and multivariate cluster analysis. The analysis was performed using the automatic statistical calculator NSSS2006/GESS2007. Statistical significance was accepted for *p* < 0.05.

## 3. Results

A total of 138 women with rUTIs and urethral syndrome participated in the study, of which 66 (47.8%) tested positive for HPV and were included in the HPV group. Table 1 shows the epidemiological characteristics. The mean age was higher in the NHPV group (48.75 years) compared to the HPV group (39.09 years, *p* = 0.001). The follow-up time was longer in the HPV group, averaging 2634 days compared to 1975 in the NHPV group (*p* = 0.0017). No differences in BMI were observed. There were also no differences between the groups regarding smoking prevalence (*p* = 1) and diseases such as depression (*p* = 0.82), diabetes (*p* = 1), or hypertension (*p* = 0.21). More urinary incontinence was observed in the NHPV group (63.89%) compared to the HPV group (18.18%) (*p* = 0.001).

The symptoms leading to urology consultation were similar in both groups. The main symptom was rUTIs, which was the reason for consultation in 60.8% of cases, predominantly in both groups: 66.67% in NHPV and 54.55% in HPV, without significant differences (*p* = 0.1649). No differences were observed regarding urethral pain, dysuria, or other concomitant symptoms. More verrucous lesions were present in the vaginal introitus in HPV patients (64%) compared to NHPV (8%) (*p* = 0.001); Table 1. 

Urine sediment analysis revealed that in the NHPV group, 8.33% of patients presented hematuria, pyuria, and ketone bodies. In the HPV group, 18% presented hematuria, 27.27% pyuria, and 9% ketone bodies. The differences in the presence of pyuria were significant, being more frequent in the HPV group (*p* = 0.001).

Vaginal exudate analysis performed at the time of urine and urethral exudate sample collection revealed that 26% of patients presented *Candida albicans*. NHPV patients had commensal flora in 25% of cases and 16.67% *Candida albicans*. In the HPV group, the proportion of patients with commensal flora was lower (9%, *p* = 0.002) and higher for *Candida albicans* (36.26%, *p* = 0.011).

The most frequent micro-organisms found in the urine cultures were *Escherichia coli* in 47.83% of cases, followed by *Klebsiella pneumoniae* in 30.43% and *Enterococcus faecalis* in 21.74%. There were differences between both groups, as shown in Table 2. All three pathogens were more frequent in the HPV group, with *Escherichia coli* found in 47.83% of urine cultures (*p* = 0.001 compared to NHPV), *Enterococcus faecalis* in 36.36% (*p* = 0.001), and *Klebsiella pneumoniae* in 21.74% (*p* = 0.001); Table 2. 

Most of the bacteria found in the urine cultures were sensitive to commonly used antibiotics such as nitrofurantoin (56.52%), trimethoprim-sulfamethoxazole (52.17%), fosfomycin (52.17%), and amoxicillin (39.13%). Few resistances were found: 21.74% were resistant to amoxicillin–clavulanate. Detailed data for each group are shown in Table 2. It should be noted that urine cultures were more sensitive in the HPV group compared to the NHPV group for amoxicillin (*p* = 0.001), cefuroxime (*p* = 0.001), fosfomycin (*p* = 0.001), nitrofurantoin (*p* = 0.001), and trimethoprim (*p* = 0.001).

When analyzing the Human Papillomavirus genotypes in the HPV group, the most common genotypes were G35 (27%), G42 (27%), and G66 (27%). The subtypes are detailed in Table 3.

In the multivariate cluster analysis, the HPV group contained the best classification and information. In the dataset analysis, the most powerful distribution variables favoring belonging to that group were antibiotic sensitivity in urine cultures: nitrofurantoin (importance of 0.96), followed by fosfomycin (0.79), trimethoprim (0.79), and amoxicillin (0.71). Other related variables, with lesser importance, were follow-up time (0.68), the presence of *Escherichia coli* (0.65), *Klebsiella pneumoniae* (0.5), and having macroscopic warts in the urethra associated with HPV (0.6); Table 4. 

## 4. Discussion

Our study analyzes the relationship between the presence of HPV in the urethra in women with rUTIs and urethral pain. The relationship of HPV as a precursor is well established in gynecological tumors and penile cancer [8]. However, its involvement in urothelial tumors is debated, with bladder cancer being where it may have a carcinogenic effect [9]. The evidence regarding its relationship with urinary tract symptoms and infections is very limited [10]. Therefore, this is the first study to explore the relationship between the presence of urethral HPV and rUTIs and urethral syndrome.

The diagnosis of rUTI requires a positive urine culture [2]. However, many women with rUTIs experience urinary tract symptoms with negative cultures during follow-up. Hypotheses have been formulated about the possible correlation between bladder pain and the microbiome, with alterations in Lactobacillus potentially presenting higher UTI rates [11]. A greater immune response in viral bladder infection has also been hypothesized, predisposing to inflammation and cystitis [10].

Our study includes 138 women who experienced these symptoms, focusing on the relationship with HPV. The study shows that more than half of the patients with rUTIs and urethral pain may have HPV in the urethra (56.5%). This population is younger but consults for clinical conditions similar to NVPH. Therefore, HPV could predispose the younger population to rUTIs compared to the older population, where factors such as estrogen deprivation have an influence, with fewer rUTIs with hormone replacement in menopausal women [2,12].

HPV induces an intracellular response, forming the basis of its carcinogenesis. The inflammatory process promotes the integration of HPV DNA, causing genomic instability [9]. Intracellular changes produce chronic inflammation that increases the production of reactive oxygen species and reactive nitrogen species, causing oxidative damage [9,13]. However, at the immune response level, the T helper response is suppressed, shifting to T regulatory cells (Treg), which have a less effective response [8].

The HPV group presented greater pyuria in the urine sediment analysis. This may be due to a chronic inflammatory state created by the virus, which could cause urinary tract symptoms, especially urethritis, as previously mentioned [2,10,11]. Additionally, a proinflammatory state in the bladder has been shown to be responsible for increased rUTIs [11]. Therefore, the presence of HPV should be studied, and its treatment through vaccines could contribute to the response in treating these symptoms (8), becoming an important new factor in this clinical condition.

Studies reveal that *Escherichia coli* is responsible for up to 80% of rUTIs, followed by lower rates of *Klebsiella pneumoniae, Enterococcus faecalis*, or *Proteus* species [14]. Our study aligns with these findings in the HPV group, with *Escherichia coli* being responsible for 81% of rUTIs. In contrast, the NVPH group shows a lower proportion of UTIs caused by *Escherichia coli, Klebsiella pneumoniae*, and *Enterococcus faecalis*. This suggests that the profile of patients with HPV presents a different bacterial profile compared to the population without it.

Several active prophylactic treatments exist for rUTIs [2], and the involvement of HPV in rUTIs could affect or modify their efficacy. The two main treatments we focus on are the prophylactic administration of antibiotics and vaccines [2].

Meta-analyses agree that antibiotic prophylaxis is the most effective treatment compared to placebo or no treatment [2,15,16,17]. However, there is growing concern about the use of antibiotics due to the resistances that arise from their administration [2,18]. In our study, we evaluated the sensitivities of the bacteria responsible for rUTIs. Bacteria in the HPV group show greater sensitivity to commonly used antibiotics such as amoxicillin, fosfomycin, and nitrofurantoin. Therefore, the presence of HPV could identify a subpopulation in which these antibiotics would be particularly effective due to the greater bacterial sensitivity observed and seen to be highly related in the cluster analysis.

Another active treatment for rUTIs is the administration of vaccines [2,7,19]. Studies have shown that the sublingual MV140 vaccine, authorized for the treatment of rUTIs [2,7], induces the production of antibodies and activates human dendritic cells to generate T helper cell (Th) 1, Th17, and regulatory T cell responses producing interleukin 10 in secondary lymphoid organs and locally in the bladder [7,20,21]. This induction of adaptive immunity is responsible for a sustained response [7]. The deregulation of the T helper response [8] in the case of HPV infection of the urothelium could reduce the vaccine’s efficacy. Additionally, HPV could, on its own, increase irritative symptoms [6]. Therefore, HPV could become a response marker, and we should investigate its role in the efficacy of treatments for rUTIs, especially immunomodulated ones. Among them, we should study the administration of HPV vaccines, which have shown high efficacy against the development of HPV [8].

The predominant HPV genotypes found in the study were G35, G42, and G66. All three types are classified as high-risk viruses for cancer development [22]. In our study, the exudate sample was taken from the urethra, where a causal association has not yet been established [23]. We need to continue investigating the field of urothelial tumors where HPV could predispose to tumor development, especially the squamous variant, as indicated by meta-analyses [9].

In this study, we employed a multivariate cluster analysis to explore the relationships between urethral HPV presence, bacterial profiles, and antibiotic sensitivity. This approach was chosen for its ability to identify natural groupings in the data without the need for prior assumptions. The analysis revealed that antibiotic sensitivity, particularly to nitrofurantoin, was the most influential factor in distinguishing the HPV-positive group, followed by fosfomycin, trimethoprim, and amoxicillin. Additional factors, such as follow-up time, the presence of Escherichia coli, Klebsiella pneumoniae, and macroscopic urethral warts, also contributed to the cluster formation.

While cluster analysis provides a deeper understanding of the data by identifying complex patterns and subgroups, it does not offer the same predictive power or straightforward interpretability as logistic regression. The “importance” values reflect the relative contribution of each variable within the cluster rather than providing direct measures of association. Therefore, these findings are exploratory and should be interpreted with caution. 

The limitations of this study are intrinsically linked to its observational design. We did not evaluate the influence of HPV on the evolution of rUTIs or its potential impact on treatment. Additionally, the definition of urethral pain has limitations as it is a diagnosis of exclusion in the absence of an active UTI, which can introduce biases in patient diagnosis. Furthermore, although the study was designed prospectively with long-term follow-up, our analysis primarily focused on the clinical and microbiological characteristics at the initial diagnosis of rUTIs. A detailed analysis of recurrences could not be conducted due to the variability in the treatments administered and the lack of complete data on subsequent cultures over the years of follow-up. This variability is also due to changes in the clinical definitions of rUTIs during the study period. We could not determine whether recurrent infections were caused by the same bacteria, as repeated cultures were not systematically available throughout the follow-up. We also cannot establish whether the presence of HPV influences the development of urological tumors, as none of the women studied developed urothelial cancer during the follow-up period. Finally, future prospective studies are needed to evaluate whether patients with rUTIs and HPV may have a differentiated response depending on the treatment administered and to better understand the dynamics of recurrences and pathogen persistence.

## 5. Conclusions

The study observed that 47.8% of women with rUTIs and urethral syndrome had HPV in the urethra. These women were younger and had longer-lasting symptoms compared to those without HPV. Additionally, a higher prevalence of certain bacteria (*Enterococcus faecalis, Escherichia coli,* and *Klebsiella pneumoniae*) was found in women with urethral HPV. However, as the study is observational, no definitive conclusions about causality can be drawn. Further research is needed to explore the role of urethral HPV in rUTIs and its impact on clinical management.

## Figures and Tables

**Table 1:** Epidemiological characteristics and consultation symptoms in women with recurrent urinary tract infections and urethral syndrome.

	NHPV n = 72		HPV n = 66		*p*-Value (*t*-Student)
Age (years), average ± SD	48.75 ± 17.57		39.09 ± 12.8		0.001
BMI (kg/m^2^), average ± SD	22.54 ± 1.25		22.76 ± 1.58		0.189
Follow-up time (in days), average ± SD	1975.35 ± 1160		2634 ± 1609		0.0017
Consultation symptoms	N	%	N	%	*p*-value (Chi-square)
ITUR	48	66.67	36	54.55	0.1649
Urethral pain	30	41.67	24	36.36	0.6014
Dysuria	18	25.00	18	27.27	0.8468
Other symptoms (Hematuria, urinary urgency)	30	41.67	24	36.36	0.6014
Warty lesions on the vaginal introitus	6	8.33	42	63.64	0.0001

SD: Standard deviation. ITUR: Recurrent urinary tract infections. NHPV: No human papillomavirus group: Patients consulting for rUTIs and urethral syndrome without urethral HPV. HPV: Human Papillomavirus group: Patients consulting for rUTIs and urethral syndrome with urethral HPV. BMI: body mass index.

**Table 2:** Distribution of micro-organisms in urine cultures * by groups. Sensitivity and resistance to antibiotics.

Groups	NHPV n = 72		HPV n = 66		Total		*p*-Value (Chi-Square)
Pathogens	n	%	n	%	n	%	
*Enterococcus faecalis*	6	8.33	24	36.36	30	21.74	0.0001
*Escherichia coli*	12	16.67	54	81.82	66	47.83	0.0001
*Klebsiella pneumoniae*	12	16.67	30	45.45	42	30.43	0.0004
*Sensitivity of antibiotics*							
Amoxicillin	12	16.67	42	63.64	54	39.13	0.0001
Cefuroxime	12	16.67	30	45.45	42	30.43	0.0004
Ciprofloxacin	18	25.00	24	36.36	42	30.43	0.1948
Fosfomycin	12	16.67	60	90.91	72	52.17	0.0001
Gentamycin	12	16.67	18	27.27	30	21.74	0.1515
Nitrofurantoin	18	25.00	60	90.91	78	56.52	0.0001
Trimethoprim	18	25.00	54	81.82	72	52.17	0.0001
Antibiotic Resistance							
Amoxicillin	6	8.33	24	36.36	30	21.74	0.0001
Amoxicillin–clavulanate	6	8.33	24	36.36	30	21.74	0.0001
Quinolones	12	16.67	6	9.09	18	13.04	0.2143

NHPV: No Human Papillomavirus group: Patients consulting for rUTIs and urethral syndrome without urethral HPV. HPV: Human Papillomavirus group: Patients consulting for rUTIs and urethral syndrome with urethral HPV. VPH: Human Papillomavirus. * Urinoculture: result of the urine culture at the time of study inclusion and simultaneous determination of urethral HPV.

**Table 3:** Human Papillomavirus genotypes in the sample.

Group	HPV n = 66	
Genotypes	n	%
G6	6	9.09
G31	6	9.09
G35	18	27.27
G42	18	27.27
G51	12	18.18
G52	6	9.09
G53	12	18.18
G61	6	9.09
G66	18	27.27
G70	6	9.09
G82	6	9.09
G89	12	18.18

HPV: Human Papillomavirus group.

**Table 4:** Multivariate cluster analysis in women with recurrent urinary tract infections and urethral syndrome: variables with the highest distribution power in the group.

Cluster	1-NHPV	2-HPV	Importance in Cluster Multivariate Analysis
Size	43.50% (n 60)	56.50% (n 78)	
Sensitivity Nitrofurantoin	Most frequent category No (90%)	Most frequent category Yes (93.30%)	0.96
Sensitivity Phosphomycin	Most frequent category No (90%)	Most frequent category Yes (84.60%)	0.79
Sensitivity Trimetropim/sulfametoxazole	Most frequent category No (90%)	Most frequent category Yes (84.60%)	0.79
Sensitivity Amoxicillin	Most frequent category No (100%)	Most frequent category Yes (69.20%)	0.71
Follow up days	Mean 1137.08 SD 1160.37 Range 28–3929	Mean 2634.00 SD 1609.017 Range 485–5421	0.68
Escherichia coli	Most frequent category No (90%)	Most frequent category Yes (76.90%)	0.64
Wart	Most frequent category No (100%)	Most frequent category Yes (61.50%)	0.60
Sensitivity Cefuroxime	Most frequent category No (100%)	Most frequent category (%)	0.50
Klebsiella pneumoniae	Most frequent category No (100%)	Most frequent category Yes (53.80%)	0.50
Ultrasound as a complementary test	Most frequent category No (80%)	Most frequent category Yes (53.80%)	0.19
BMI	Mean 22.54 SD 1.25 Range 20.82–24.38	Mean 22.76 SD 1.58 Range 19.15–24.38	0.11
Age	Mean 48,75 SD 17,578 Range 22–73	Mean 39,09 SD 12.80 Range 24–63	0.9
Positive urine culture	Most frequent category Yes (60%)	Most frequent category Yes (61.50%)	0.1

NHPV: No Human Papillomavirus group: Patients consulting for rUTIs and urethral syndrome without urethral HPV. HPV: Human Papillomavirus group: Patients consulting for rUTIs and urethral syndrome with urethral HPV. VPH: Human Papillomavirus.

## Data Availability

Data is available under reasonable request contacting with authors.
